# Genomic surveillance urgently needed to control wheat blast pandemic spreading across continents

**DOI:** 10.1371/journal.pbio.3002090

**Published:** 2023-04-12

**Authors:** Johanna Rhodes

**Affiliations:** Department of Medical Microbiology, Radboudumc, Nijmegen, the Netherlands

## Abstract

This Primer explores a new study in PLOS Biology which describes the alarming potential of a pandemic clone of wheat blast disease to evolve fungicide-insensitive variants, arguing the urgent need for genomic surveillance and pre-emptive breeding of resistant wheat.

## Teaser

A new study in *PLOS Biology* highlights the alarming potential of a pandemic clone of wheat blast disease to evolve fungicide-insensitive variants and argues the urgent need for genomic surveillance and preemptive breeding of resistant wheat.

Our dependence on wheat is high; wheat is the staple food for 35% of the world’s population, and 25% of the wheat produced globally is used for livestock feed and industrial uses. Yet yield losses caused by pests and diseases average over 20% [1]. Worryingly, a blast disease caused by *Magnaporthe oryzae* has the capacity to create a pandemic, creating further losses and resulting in global food insecurity.

**Fig 1:**
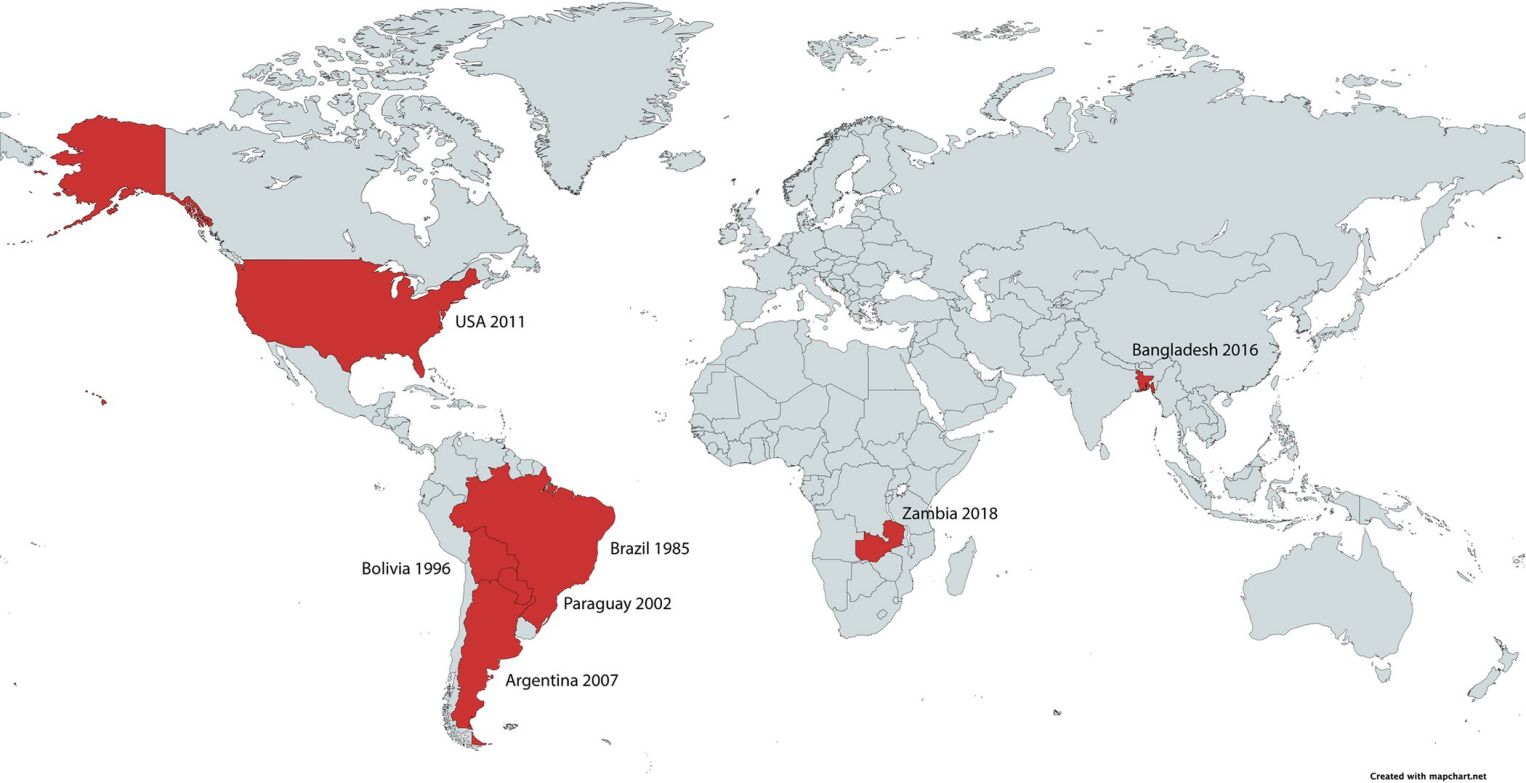
Global appearance of wheat blast disease (adapted from [2] and created with www.mapchart.net).

Wheat Blast was initially identified in Brazil in 1985 and has subsequently spread to major wheat-producing areas, as well as other South American countries, Bangladesh, and Zambia via international trade (Fig 1) [2]. In its wake, Wheat Blast has left catastrophic crop losses: Bolivia has recorded a 69% crop loss; in the Southern Cone region of South America, yield losses up 100% have been recorded; and in 2016, outbreak in Bangladesh reduced yield by up to 51%. It is clear to see, then, that further spread of Wheat Blast would cripple world food security.

Genomic surveillance presents an opportunity to provide important information for the timely identification of this disease and tracking its spread. The World Health Organization have recently released their global strategy for global genomic surveillance of pathogens with pandemic and epidemic potential [3]. Wheat Blast is a prime candidate for genomic surveillance, yet this would be challenging. Rapid, effective surveillance would be dependent on quick and accurate identification of the pathogen “in the field,” coupled with extensive and boundary-less data sharing and analysis. The SARS-CoV-2 pandemic has shown we are capable of establishing such surveillance networks (e.g., COG-UK), and while their formation are not trivial, we are yet to see such networks developed for fungal diseases.

Latorre and colleagues argue the urgent need for genomic surveillance to track and mitigate the spread of Wheat Blast outside of South America and have brilliantly demonstrated the utility of genomic data for epidemiological surveillance of Wheat Blast [4]. By selecting a discriminate set of markets from whole genome sequence data (84 SNPs), they confirm that a clonal lineage of Wheat Blast, B71, has spread on two independent occasions from genetically diverse South American populations to Zambia and Bangladesh and has pandemic potential. A global genomic surveillance system would therefore improve tracking and monitoring of Wheat Blast, enabling the identification of variants of concern soon after they emerge. This would subsequently enable implementation of management strategies, such as containment or quarantine, or “wheat holidays” where wheat cultivation in Wheat Blast–affected or vulnerable area is suspended [2].

Yet the value of the genomic data generated by Latorre and colleagues has been in the identification of effectors that can be targeted by the plant immune system. Since plant pathogens secrete effectors to cause infection, the host has used this same system to trigger plant immunity through avirulence activity. Previous research efforts have shown that the most predictive avirulence effector for Wheat Blast resistance is AVR-Rmg8, which carries the resistance gene *Rmg8* and subsequently creating an immune response in the host [5]. By sequencing the genomes of pandemic B71 isolates, Latorre and colleagues have shown that these clonal strains are incapable of infecting wheat plants with *Rmg8* because AVR-Rmg8 is conserved within this particular lineage. This offers a rare and promising opportunity to prevent massive food insecurity by breeding and distributing B71-resistant wheat varieties that carry *Rmg8* to high-risk areas. Yet, as we are constantly in an “arms race” with pathogens, it is likely Wheat Blast would eventually evolve virulent strains. Therefore, breeding programs could combine *Rmg8* with additional sources of resistance, such as other resistance genes including *Rmg2*, *Rmg3*, *Rmg7*, and *RmgGR119* [2]. Resistance, or “*R*” genes with broad spectrum resistance, should also be considered. With the accumulation of more whole genome sequences, genome-wide association studies will also identify potential loci for Wheat Blast resistance.

Future breeding programs could utilize the versatile CRISPR/Cas genome editing systems in short time frame. The Cas9 system for DNA modification has recently been used to enhance disease resistance in rice against rice blast disease caused by *M*. *oryzae* [6], suggesting similar potential for wheat, too. More recently, the RNA editing Cas13 system also provides an avenue to explore to generate Wheat Blast–resistant wheat varieties [7]. However, these genome editing systems also enable manipulation of the *M*. *oryzae* genome, creating mutants with reduced growth, differential pathogenicity, or altered germination qualities. Ultimately, more long-term strategies should look into plant disease management via the targeting of plant disease resistance mechanisms as well as the potential for fungal genome editing using CRISPR/Cas systems. These strategies could be preferable to more extreme measures, such as quarantine or border control to prevent the spread of fungi via trade routes, which would ultimately disrupt the market and the capacity to create a spike in food prices.

While breeding and surveillance strategies may be more long-term solutions, in the short term, B71 isolates were also seen to be sensitive to strobilurin fungicides. However, we cannot heavily rely on fungicide treatment to mitigate the spread of the pandemic lineage, which is at risk of developing resistance via a nonsynonymous SNP causing a glycine to alanine shift (G1243C) in Cytochrome B (*CYTB*). Wheat Blast isolates are also capable of mating with prevailing finger miller blast isolates, which would potentially create more genetic diversity and drive the evolutionary potential of this pandemic lineage.

It is clear that *M*. *oryzae* is evolving quickly and could cause potentially disastrous crop losses and failures in various regions of the world, if its spread is allowed to continue. In order to prevent global food insecurity, it is vital we heed the findings in Latorre and colleagues and work together (as highlighted by their efforts through the OpenWheatBlast Community) to create a global effort to prevent any further destruction.
